# Introduction and integration of PrEP and sexual and reproductive health services for young people: Health provider perspectives from South Africa

**DOI:** 10.3389/frph.2022.1086558

**Published:** 2023-01-09

**Authors:** Melanie Pleaner, Fiona Scorgie, Catherine Martin, Vusile Butler, Lorrein Muhwava, Maserame Mojapele, Saiqa Mullick

**Affiliations:** Wits RHI, Faculty of Health Sciences, University of the Witwatersrand, Johannesburg, South Africa

**Keywords:** oral PrEP, health providers, health systems, HIV prevention, sexual and reproductive health (SRH), integration, adolescent girls, young women (AGYW)

## Abstract

South Africa has one of the largest HIV epidemics in the world, with particularly high prevalence among adolescent girls and young women (AGYW). Oral PrEP was introduced in the public sector in 2016 in a phased manner. Given the important role played by health providers, research was undertaken to understand their experiences of and attitudes towards introduction of PrEP as a new HIV prevention method, and its integration within broader sexual and reproductive health (SRH) services for youth. A survey was undertaken with 48 purposively sampled health providers working in primary health care facilities and mobile clinics in three provinces in South Africa. Qualitative analysis was performed on free-text responses to open-ended questions in the survey, using an inductive approach to code the data in NVivo v.12 software. Health providers expressed concerns about adding a new service to an already overburdened health system, and worried that young people seeking PrEP would divert staff from other critical services. While most recognised the benefits and opportunities afforded by HIV and SRH service integration, providers highlighted the extra time and resources such integration would require. Many were anxious that PrEP would encourage disinhibition and increase unprotected sex among AGYW, and held judgemental attitudes about young people, seen as largely incapable of taking responsibility for their health. Findings underscore the importance of consulting health providers about implementation design and providing channels for them to express their misgivings and concerns, and training needs to be designed to address provider attitudes and values. Opportunities need to be sought to strengthen the provision of adolescent and youth friendly services—including adolescent-health provider dialogues. Insights from this study can assist in guiding the introduction of new HIV prevention methods into the future.

## Introduction

South Africa has one of the largest HIV epidemics in the world, with 38% of new infections in 2017 occurring among youth aged 15–24 years. Prevalence among adolescent girls and young women (AGYW) in this group is particularly high: nearly four times greater than that of young men (1, 2). This disparity is largely driven by cross-cutting factors such as poverty and unemployment, age disparate sex (3, 4), and transactional sex (4, 5), against a background of harmful gender norms and unequal gender power dynamics, where the rights, safety and choices of AGYW are severely compromised (6, 7).

Oral pre-exposure prophylaxis (PrEP), comprising the antiretrovirals emtricitabine (FTC) and tenofovir disoproxil fumarate (TDF), has provided a much-needed HIV prevention option globally, and is over 90% effective when used correctly (8). Oral PrEP was introduced into South Africa in 2016 in a phased manner, with initial provision in demonstration and study sites, followed by expanded provision to sex workers, men who have sex with men, and AGYW project sites. It was subsequently rolled out to include public sector primary healthcare clinics, and is supported by national Department of Health guidelines (9).

Health providers play a critical role in the introduction and promotion of new services and are recognised as important catalysts for change. Indeed, their willingness to make adaptations to existing services and their creativity in integrating appropriate changes may determine the success or failure of new health technologies. But health providers may lack the necessary motivation to introduce new methods. Among other factors, this may result from a misalignment between professional roles and expectations on the one hand, and personally held values on the other (10).

Indeed, the introduction of new products is not always supported by the requisite health systems. We can draw lessons from the introduction of other interventions within the public health sector—such as the early introduction of antiretroviral treatment and the subdermal contraceptive implant. Similarly, as PrEP is scaled up in national programmes around the world, lessons are being learnt about the need for robust and appropriate health systems required to support quality of care to ensure optimal health benefits for adolescent clients (11, 12).

Encouragingly, integration with sexual and reproductive health (SRH) services has been shown to improve effective use of PrEP (11), in addition to generating other benefits, such as creating opportunities to promote both HIV and SRH services (13). From a health provider's perspective, integration brings expanded skills development, improved job satisfaction, a reduction in workload (14), improved efficiency, and increased staff motivation (15).

Health providers may feel ambivalent about providing integrated services, however. A study from Kenya looking at health providers’ experience of integration of HIV and SRH services identified a number of challenges. These included poor health systems to support service delivery, limited physical space, equipment, drugs and other medical supplies, and perceptions of increased occupational stress and workload (15). Training, capacity building and ongoing support for health providers were found to be crucial components of the health systems strengthening needed to improve health providers’ performance and attitudes.

There is growing recognition of the need for HIV and SRH programmes to be responsive to the needs of young people (16, 17) and—given their heightened vulnerability—to AGYW in particular. Substantial work has been undertaken to define what constitutes quality of care for adolescent and youth friendly services (AYFS) (12, 16, 18). There is global consensus that the cornerstone of AYFS is the provision of services which are accessible, acceptable, equitable, appropriate, and effective, with a key focus on the provision of evidence-based, non-judgmental and non-discriminatory care (18, 19). Again, health providers’ attitudes are key: if these attitudes discourage young people from accessing PrEP and other SRH services, this will directly impact on service utilisation and health outcomes (10, 20–22). South Africa has generated standards and tools to benchmark quality AYFS, which foreground staff attitudes as critical to the successful provision of these services (23–25). A recent evaluation of facilities in two health sub-districts in the country found that overall, they failed to meet many of the criteria for youth-friendly service provision, suggesting that we still have a long way to go in this regard (26).

Notably, it is in this institutional context that PrEP is being introduced in South Africa, as an additional female-controlled option to prevent HIV (11). PrEP has been an important breakthrough for AGYW and has formed the centre-point of several national AGYW-focused programmes in the country, such as “She Conquers” (27) and DREAMS (28). Yet there is limited evidence on implementation strategies for providing PrEP to AGYW as part of an integrated package of AYFS (29).

In this article, we analyse health providers’ views and experiences of the introduction of PrEP into integrated, adolescent- and youth-friendly HIV and SRH services, as well as their perceptions of PrEP as an appropriate HIV prevention method for young people and for AGYW in particular. We draw on data from the Unitaid funded “Project PrEP”, which hosted this research. Implemented since December 2018, the project aimed to identify and develop models of integrated service delivery for quality HIV and SRH services, with a focus on AGYW. It generated real world evidence on the introduction and integration of PrEP as it was being rolled out in primary healthcare (PHC) clinics and mobile services in four provinces of South Africa.

## Materials and methods

### Study design

Qualitative data for this analysis were collected as part of Project PrEP's exploration of the perceptions and experiences of health providers in relation to the introduction of PrEP into comprehensive services for AGYW in South Africa. We analysed free-text responses to open-ended questions in a semi-structured survey undertaken between February 2019 and May 2020 with health providers working in participating PHC facilities and mobile services. The survey coincided with the National Department of Health (NDoH) roll-out of PrEP in public health clinics, which targeted people at substantial risk of HIV infection, including AGYW. At the time of data collection, many of the sites had only just begun to provide PrEP and some health providers had not yet been trained.

### Setting

South Africa's health system is two-tiered and highly unequal. The underfunded public sector is accessed by the majority of the population, which largely cannot afford the health insurance needed to access the well-resourced private sector (30). Primary care service provision in the public sector is dominated by chronic long-term care (HIV and non-communicable diseases), with additional service streams focused on acute care (minor ailments), preventive and promotive services (maternal and child health and SRH) and health support services. Although service utilisation may vary by site and depending on the local burden of disease, research among primary care facilities in Kwa Zulu Natal has indicated that in 2020, clinic visits for ART follow-up care accounted for almost half of all clinic visits (43%), followed by visits for minor ailments (18%), child health (11%) and hypertension (10%) (31).

The study was undertaken in four diverse (urban, peri-urban, and rural) geographical clusters in three provinces in South Africa (Gauteng, KwaZulu-Natal and Eastern Cape). Each project cluster consists of two fixed-site, primary care facilities and a project mobile clinic to extend the reach of services within the surrounding community. Participating facilities offer a range of integrated services for AGYW, including HIV testing, contraception, sexually transmitted infection (STI) and PrEP services, and linkages to HIV treatment services as required. Project facilities were selected based on their burden of HIV, teenage pregnancy, STIs and gender-based violence, as well as their proximity to secondary and tertiary educational facilities where adolescents and youth may be reached.

### Recruitment and sampling

Health providers were purposively sampled from the eight participating facilities and four mobile services, based on their expertise, experience and role in planning the PrEP roll-out or in providing SRH or PrEP services to adolescents. Only health providers working in the project sites and willing to consent to the survey and to administration of the survey being audio-recorded were eligible for recruitment. Participants were eligible irrespective of gender, health provider cadre, or number of years of experience.

The study team worked with facility managers, who suggested other eligible healthcare providers within their facility who could be invited to participate in the study. Participants were recruited face-to-face by members of the project team. We also made use of snowball sampling among recruited participants to identify additional participants and build a sample large enough to obtain a diversity of views. Some participants were interviewed twice, in order to capture reflections on the implementation process after the roll-out. Recruitment continued until data saturation was reached, at which point 48 participants had been interviewed across the study sites.

### Data collection

Surveys were administered face-to-face and telephonically (the latter for participants who were too busy to meet in person and when COVID-19 restrictions were in place in 2020). Interviewers were trained in research ethics and in the skills required to conduct high-quality, reliable surveys, including how to handle open-ended questions. The survey was administered in the language of participants’ choice (English, isiZulu or seSotho), and open-ended responses were audio-recorded. Participants were assigned a unique number to ensure anonymity when identifying themselves for the audio recording.

The open-ended questions in the survey (roughly one-fifth of the tool) focused on a range of topics including: training received on PrEP provision, demand creation strategies, uptake and consistent use of PrEP, major programme challenges and successes, and lessons learned in providing integrated services to adolescents and youth.

### Data analysis

Descriptive analysis of demographic data was conducted. Audio recordings were translated into English, where necessary, and transcribed verbatim for analysis. A team of three analysts open-coded the transcripts using NVivo software (version 12, QSR International, Melbourne, Australia), using an inductive coding approach influenced by Grounded Theory (32). Analysts consulted with one another throughout the process to build consensus on the coding framework and ensure consistent application of codes. Key themes emerging from the coding were identified and further developed through the writing of detailed coding summaries, which formed the basis of the manuscript.

### Ethical approval

The study was approved by the Human Research Ethics Committee at the University of the Witwatersrand (M180806) and by the World Health Organization (WHO) Ethics Research Committee (Wits-PrEP-AGYW). All participants provided written informed consent before taking part in the interviews.

## Results

A total of 48 health providers participated in the interviews. The majority were female (93.7%) and professional nurses (68.7%), with the remainder consisting of doctors, lay counsellors and administrators. This gendered and professional bias is largely reflective of the fact that in South Africa, health providers are mostly female, especially at nurse level, and that PrEP services at PHC facilities are generally provided by professional nurses (31). Table 1 summarises key demographic characteristics of these participants.

**Table 1:** Demographic characteristics of the sample of health providers (*n* = 48).

Variable	*n* (%)
Gender	
Female	45 (93.7)
Male	3 (6.2)
Age group (years)	
26–35	14 (29.1)
36–45	17 (35.4)
46–55	8 (16.6)
56+	9 (18.7)
Years of experience*	
<5	4 (8.5)
6–10	17 (36.1)
11–15	7 (14.8)
16–20	6 (12.7)
20+	13 (27.6)
Health care provider category	
Professional nurse	33 (68.7)
Lay counsellor	10 (20.8)
Medical doctor	2 (4.1)
Admin staff	3 (6.2)
Geographical location	
Urban formal	40 (83.3)
Rural	8 (16.6)
Providing PrEP at time of survey	45 (93.7)

* Data for 1 participant missing.

Our analysis of the interview data identified three broad themes in health providers’ perspectives on the PrEP roll-out. These included views on (1) challenges, concerns, and anticipated benefits of integrating PrEP into existing SRH services for young people; (2) health system requirements to support PrEP delivery; and (3) attitudes towards PrEP as an appropriate HIV prevention method for youth, and for AGYW specifically.

### Introducing PrEP as an integrated service: Challenges and benefits

When health providers were asked how they felt about absorbing the provision of PrEP into existing services, many described PrEP as an extra, add-on service that was problematic in a context where the health system is already overburdened. There was concern that HIV prevention services were receiving priority over other services, and some confusion about where PrEP services should ideally be located within the clinic structure.

Participants complained about heavy caseloads of ill clients and saw PrEP-users as an additional burden on a health system that could not absorb any more clients—especially clients who are fundamentally well, and not “*sick*” or requiring treatment. This was a view that was apparently held by many of the participants’ colleagues as well. Reflecting on what she saw as an increased workload for health providers, one participant complained, “*it's too much, I don't want to lie*” (42-year-old female professional nurse, Eastern Cape). Another participant admitted:

*It’s a bit scary and daunting, because as you say the training won’t last forever…at some point we will have to start rolling out ourselves and obviously it’s gonna be stats and a lot of paperwork. It’s very daunting, it’s like an additional workload on our HIV programme* (33-year-old female professional nurse, KwaZulu-Natal).

Some had observed a growing demand for PrEP that was proving difficult to manage. One participant in KwaZulu-Natal said, “*They (youth) are bringing their people for PrEP. I am overwhelmed*” (43-year-old female professional nurse, KwaZulu-Natal).

A number of participants from facilities where the PrEP roll-out was well underway claimed that PrEP service provision was impacting negatively on other essential services. Participants in Gauteng spoke of how other programmes in the clinic “*are suffering*” because health providers are expected to focus on PrEP initiation. In sites where PrEP was already being provided, there were complaints that PrEP was diverting nurses from other PHC services. This problem was said to be further exacerbated by the regulatory requirement in South Africa that only nurses trained as NIMART (Nurse Initiated Management of Antiretroviral Therapy) providers are allowed to administer PrEP. A NIMART nurse in Gauteng complained that she was regularly called away from ART services to deal with initiating new PrEP users: “*We have a shortage of staff as it is. So, for people to leave whatever they are doing, to come and assist with PrEP is very difficult…*” (43-year-old female professional nurse, Gauteng). Similarly, an Eastern Cape participant working as a lone nurse in antenatal care (ANC) described how her ANC clients were frequently left waiting while she was called away to initiate other clients onto PrEP.

There was uncertainty about how to classify PrEP users within the spectrum of clients accessing their facilities—and therefore how to position PrEP services in relation to existing services. Conflicting views were expressed about offering PrEP as an integrated versus a vertical service, and concern about how this decision may impact on PrEP uptake and quality of care.

*Are they [PrEP clients] chronic or acute? So where do they stay? Even if they come to the clinic, they are the priority, or they must follow the line [queue]? That’s where we will lose them. If they follow the long queues yet they know that they came for PrEP only, that’s how we can lose them* (43-year-old male professional nurse, KwaZulu-Natal).

Some health providers felt that PrEP services should be offered by a dedicated health provider—separate from PHC services—to ensure that PrEP clients do not swamp the already long queues. Rather than reducing waiting times, there was a feeling that integration had the opposite effect. One participant in Gauteng pointed out that “*clients are in a hurry, [and integration] requires longer consultation times*” (40-year-old female lay counsellor, Gauteng). Another regarded the comprehensive counselling needed at PrEP initiation as too time-consuming:

*You have to educate and counsel, counsel, counsel. To say [to clients], “these are the disadvantages of not taking [PrEP]” – that takes time. [Meanwhile], the ones outside are complaining* (49-year-old female professional nurse, Eastern Cape).

Numerous participants felt that a range of training, skills and experience was needed for health providers to render integrated services—in addition to the PrEP-specific training that accompanied the roll-out. There were additional concerns about whether facilities could support the one-stop-shop concept when aspects of the service were still fragmented. For example, clients could receive PrEP tablets directly from a provider but if prescribed STI treatment they still had to queue up at the dispensary for the latter.

Overall, however, most health providers in the study supported integration of PrEP with other services and saw the “one-stop shop” approach as reducing waiting times, among other benefits. Some pointed out that dealing with PrEP “*in isolation*” would in fact be challenging since clients themselves bring multiple issues to their consultations. Furthermore, it was believed that referral to multiple health providers for different services—as necessitated by vertically arranged services—would become a barrier for young people accessing PrEP. As one provider put it, “*Youth don't like going around*” (29-year-old female peer educator, Gauteng).

Those who supported integrating PrEP into existing SRH services recognised that this would provide more opportunities to reach young people and improve their health outcomes, especially in relation to averting unplanned pregnancies, identifying and managing STIs, increasing HIV testing and promoting dual protection and condom use. PrEP integration could therefore be an opportunity to go beyond HIV prevention and improve uptake of other SRH services, in other words. One nurse claimed:

*I always advise people who are on PrEP to use one of the family planning methods. I always encourage everyone, and say: “Are you sexually active?” They say, “Yes”… “Are you using contraceptives?” “No, I’m not”… I give them all the options… they leave with PrEP and family planning. And my family planning stats are increasing because of that* (43-year-old male professional nurse, KwaZulu-Natal).

As this quote suggests, the beneficial knock-on effects of PrEP integration were already becoming evident in some facilities. One nurse reported that since offering PrEP as a service integrated with broader SRH services in her facility, there has been an increase in uptake of HIV testing, which in turn has increased ART initiation. Some providers also favoured integration of PrEP and ART services as a strategy that would help to de-stigmatise HIV services: “*It won't be clear if you are a [HIV] positive somebody or you are a negative somebody, [because] everything will be in the same room*” (34-year-old female professional nurse, KwaZulu-Natal).

Other potential benefits of integration identified by participants included the protection of client privacy, which was deemed to be especially important for young people, who are at risk of stigma if seen by other clients to be accessing SRH services. Furthermore, when clients were not referred to multiple members of staff, they could build trust with a single health provider instead—something that was regarded as essential when dealing with SRH and HIV.

Questions about offering PrEP as integrated versus vertical service surfaced again in discussions about the wisdom of integrating the PrEP roll-out with adolescent and youth friendly services (AYFS). In facilities where there is no dedicated youth-specific zones or rooms, participants pointed out that young people must join the often long queue for clients seeking PHC services. In addition to eschewing referral to multiple providers, it was said that young people also “*don't want to wait*” (38-year-old female professional nurse, Eastern Cape). Participants were concerned that long queues would be even more of a deterrent in the case of PrEP, because the population seeking PrEP are generally healthy:

*Maybe the youth will end up saying, ‘since I am not positive for now, I was just protecting myself, then let me go and come back another day’* (34-year-old female professional nurse, KwaZulu-Natal).

One health provider, who expressed a preference for a stand-alone, prefabricated set of rooms for adolescent and youth services, explained that this separate space would not only remove healthy PrEP clients from the PHC queue, it would also avoid a situation where “*other clients will complain that ‘why are PrEP clients coming in and being served before us?’*” (27-year-old female professional nurse, Gauteng).

In other words, the impetus to offer separate services for young people was borne out of a desire to avoid swamping the clinic queues and not necessarily from a recognition that young people want discretion and privacy when seeking sexual health services. This, in turn, motivated some healthcare providers to call for PrEP to be provided as a stand-alone service rather than being integrated into PHC, with dedicated PrEP staff in a youth-friendly environment.

### Health systems required to support the provision of PrEP services

A key focus of our inquiry was to explore views relating to aspects of the health system which need strengthening to support the delivery of PrEP and integration, and where the gaps may be in this regard. A number of participants expressed concerns about the ability of the South African health system to support PrEP services, pointing to likely challenges with access and coverage, training, systems to support drug storage and supply, and demand creation.

Notwithstanding the existence of mobile clinic services, many health providers felt that distances from and transport costs to clinics, and the limited scope of the roll-out could deter those seeking PrEP services. Staff shortages in some facilities were seen as potentially affecting PrEP access, particularly in the afternoons when fewer staff are on duty—at precisely the time when youth tend to access services, after school hours.

Health providers across all provinces identified lack of staff training as a potential barrier to PrEP uptake. In some facilities only some staff members had been trained; this was linked to weak buy-in and commitment from staff to embrace PrEP. One nurse reflected on her experience of this kind of uneven training:

*It is just me who attended [PrEP training] and the professional nurse, you know. It is not the majority of them – in such a way that when it comes [to providing PrEP to clients] they are having some doubts, to say “Do we really? Are we supposed [to provide PrEP]?* (59-year-old female professional nurse, Gauteng).

It was felt that there was a danger that the one PrEP-trained provider in a facility would become labelled as the “*PrEP nurse*”, thereby removing the incentive for untrained staff to sign up for training or inform themselves about PrEP. One provider in the Eastern Cape believed this should be avoided at all costs:

*If all the professional nurses in this section were trained, they would easily give the information to the clients without sending them to somebody else, you see* (58-year-old female professional nurse, Eastern Cape).

Aside from underlining the value of integration of services, her comments raise the question of how respective roles and responsibilities within the provision of PrEP are outlined to the staff team. The need for all staff members in a facility to receive at least some PrEP orientation was considered essential but appeared to not be happening in practice. Indeed, it was not always clear who was responsible for the training of staff, as a DoH training plan for PrEP introduction was expected but did not always materialise. DoH training of more NIMART nurses was specifically requested, but there was also a general call for more comprehensive training of health providers, covering not only the clinical provision of PrEP, but also the integrated provision of other SRH services:

*… the only challenge will be if we don’t have clinicians…who are having all [skills], like NIMART, family planning, SRH. So, you need to have somebody who has all these small…skills* (59-year-old female professional nurse, KwaZulu-Natal).

Additional health system weaknesses that participants highlighted as relevant for PrEP provision revolved around space and infrastructure in clinics, which had implications for issues such as privacy, multiple service delivery points, waiting times, and drug storage. This latter point was an important consideration for PrEP, which was described as medication “*to be kept under a lock and key… in a safe place*” (43-year-old female professional nurse, Gauteng). Furthermore, health providers’ experience with contraceptive commodity shortages raised similar concerns about possible stock-outs of PrEP.

Finally, there was recognition that a successful PrEP roll-out required intensive demand creation efforts. Several health providers expressed a desire to do more demand creation but complained that there was not enough time available for it. Two participants in KwaZulu-Natal lamented the fact that demand creation was beyond their reach. One said, “*we don't have dedicated staff members that can do school health, that's where we can identify these teenagers*” (55-year-old female professional nurse). The other, a 34-year-old female professional nurse in a nearby facility, said,

*We don’t get enough time to talk to them… in the clinics it’s just, you have to work, work, work. So, if maybe we had an opportunity to go to schools, universities, as our target market is [there], they could allow the nurses to go and initiate PrEP. That’s the only barrier because once they know [about PrEP], they start*.

### Health provider attitudes toward offering PrEP to young people

A fundamental aspect of the provision (and integration) of PrEP to maximise access for young people is the nature of health providers’ attitudes towards young clients. We were interested to know whether healthcare providers saw PrEP as an appropriate HIV prevention method for youth and for AGYW specifically, what kinds of attitudes participants had observed among their colleagues and how these were affecting service provision.

Some participants were somewhat defensive, claiming that young people tended to misunderstand health providers’ responses to them and often incorrectly assumed that they would be met with hostility.

*Eish, our youth… (laughs), when they see nurses… they think nurses are rude and all that… they are perceiving nurses as those horrible people.* (43-year-old female professional nurse, Gauteng).

*I think ya, they are afraid because of that myth about nurses, that nurses are rude. Some of them become scared to come to us* (28-year-old professional nurse, Eastern Cape).

A larger proportion of participants recognised that many healthcare providers were indeed judgemental of young people, however, and recognised how this would become a barrier to services. There were multiple descriptions of how colleagues in their facilities displayed indifference to the needs of the youth, at best:

*[Some healthcare providers] just say “aah, nxh! I don’t care about them. Let’s go that side, these kids are troublesome”… That is your attitude towards PrEP and the youth. But if you have a positive attitude, you will even call them as they walk there, [saying] “come, come, come”* (55-year-old female professional nurse, Gauteng).

Concerns were raised about how negative staff attitudes could impact on both service utilisation and effective PrEP use. Describing one young woman who started PrEP but did not return to the clinic for follow-up appointments, a peer educator said,

*A few months later she came back and when you ask, “why you stopped PrEP?”, they say “because of the attitude that I get here, [it] makes me afraid to come back. I was not treated fairly”* (29-year-old female peer educator, Gauteng).

Some health providers spoke about colleagues who are actively against PrEP, saying they “*don't wanna know about it*” and there were even accounts of clients asking for PrEP but being chased away by health providers. Others speculated that the reason for ‘anti-PrEP’ attitudes among health providers may be a lack of training and knowledge, or even an underlying suspicion about the motives for PrEP introduction:

*I think the other health care providers, they are incompetent in terms of PrEP. They have this mentality that has negative thoughts behind PrEP. They are not sure, they think this drug is just there for statistics, money-wise something, something, somebody will benefit at the end”* (30-year-old female professional nurse, KwaZulu-Natal).

A number of participants had much to say about the kind of approach health providers should take with young clients, and the need to communicate in a non-judgemental and youth sensitive manner. As one participant put it:

*“When you are facing young people, you [should] take your mind, your brain away, your grudges and your elderly mind, and you just go down and meet the needs of this person.”* (63-year-old female professional nurse, Eastern Cape)

Despite the willingness to call out judgemental staff attitudes and an apparent familiarity with the principles of providing AYFS, these same participants at times readily criticised young people and were openly dismissive of their ability to practise safe sex. In part, this reflected a deeper scepticism about the wisdom of supplying young people with PrEP. A large majority of healthcare providers interviewed considered the behaviour of young people to be inherently problematic because of their lack of self-efficacy to take responsibility for their own health. One participant exclaimed, “*Ya! Adolescents can be dumb!*” when this issue came up in the interview. Young people were described as “*irresponsible*”, “*careless*”, and “*reckless*”, routinely engaging in “*sexual unruly behaviour*”, which many participants believed would increase once they start taking PrEP. Young people were characterised as having a “*mentality in them that is not mature enough*” for stable relationships (42-year-old female professional nurse, KwaZulu-Natal). A number of participants labelled youth “*promiscuous*”, saying that they were “*just sleeping around*”—a characterisation that was implicitly gendered, as it turned out that they were in fact referring to AGYW. On the subject of risk and sexual behaviour, for example, one provider said:

*“They will do it. They will have unprotected sex. They did that even before PrEP came, for money and when they are with their partners. They cannot stand firm and ask for a condom”* (55-year-old female lay counsellor, KwaZulu-Natal).

A dominant view in the interviews was that PrEP use would inevitably lead to sexual disinhibition among AGYW. A number of participants—both young and older—were concerned that giving PrEP to AGYW at clinics would be seen as effectively “*legitimising*” sex and offering them “*a free pass*” (26-year-old female medical doctor, Eastern Cape). A common perspective on this matter was the belief that:

*…they will go, knowing that “I’m taking this PrEP medications so I can sleep around whatever without getting infected”* (43-year-old female professional nurse, Gauteng).

There was concern that condom use would decline, while several health providers worried that PrEP usage would be followed by an increase in STIs and unplanned pregnancies.

*“It will increase the rate of unplanned pregnancies. As it is, they come to the clinic, already pregnant from all these universities. So, if they take PrEP, and not condomise as we advise them to, they will continue getting pregnant”* (55-year-old female lay counsellor, KwaZulu-Natal).

One provider believed that young people would even go so far as to deliberately indulge in risky sex purely to “*test*” the efficacy of PrEP, and thus to “*test*” the credibility of health providers themselves.

*“They will actually want to test whether the healthcare providers are lying or are they telling the truth that I cannot get HIV by using PrEP”* (38-year-old female professional nurse, Gauteng).

A common explanation for this risk-taking behaviour was that youth were “*not well informed*”, and consequently, more education was the only way to remedy the situation. There were calls for “*continuous education, continuous counselling*” (59-year-old female professional nurse, Eastern Cape) to encourage adherence, together with motivational strategies to encourage behaviour change: “*We must counsel them and motivate them to use condoms all the time*” (46-year-old female lay counsellor, Eastern Cape).

Only a small minority of participants expressed more positive views of how PrEP may be empowering for young people, giving them a tool to protect themselves against HIV. One health provider said that PrEP will make young people feel “*very proud*” of themselves, that they will feel the same as if they had “*got a new job or had bought new shoes*” (63-year-old female professional nurse, Eastern Cape). A second health provider challenged the popular, moralistic belief that health technologies could lead to disinhibition of the very behaviour they are designed to prevent, drawing an analogy with contraception myths:

*At the beginning, a long time ago, there were people [who] were not accepting family planning methods, saying it encourages people to promiscuity, you see! So, it [PrEP] can be the [same] concept as that…I don’t think that it can, [that] it will allow them to do that [behave promiscuously]* (59-year-old female professional nurse, Gauteng).

Ultimately, however, young people were presented as fundamentally incapable of using preventative technologies, with responsibility for this ‘failure’ resting with youth themselves—as one put it, “*it’s the failure of youth to know what is right for them*” (34-year-old female professional nurse, KwaZulu-Natal)—rather than with the technologies or with the unsupportive environments in which they are introduced.

## Discussion

Health providers are at the heart of service delivery and their perspectives on the barriers and enabling factors that support or hinder the provision of quality, integrated youth-friendly PrEP and SRH services are therefore important to understand. This study provided in-depth, granular insights into issues that need to be considered when planning for new prevention methods to become part of integrated services. We found that health providers in rural, peri-urban and urban facilities across three provinces in South Africa were ambivalent about integrating PrEP with SRH services. Our findings echoed those of other studies on the topic of HIV and SRH services integration—in which health providers have supported integration as beneficial but also had misgivings about the possibility of extra work (11, 14, 15).

On the one hand, health providers in our study recognised a certain logic to PrEP being offered as part of this integrated package—given the interconnectedness of clients’ HIV and SRH needs—and believed such integration would benefit both health providers and clients. On the other hand, providers expressed uncertainty and confusion as to where PrEP fits into the PHC and the HIV care continuum, whether PrEP should be integrated or provided as a vertical service, and how existing staffing arrangements will affect facilities’ capacity to absorb this new service. When introducing PrEP services at site level, there is a need for careful engagement with both management and frontline workers on issues such as points of entry for clients, location of PrEP services within clinic structures, and staff training. Health providers need opportunities to express their concerns regarding the additional capacity, time and resources required to integrate PrEP into existing services.

The challenges facing Nurse Initiated and Management of Antiretroviral Treatment (NIMART) trained nurses has been well documented (33–35). The Department of Health pharmaceutical guidelines for the provision of PrEP are the same as for ART (Schedule 4), and as such, require NIMART nurses to be available for the provision thereof (9). As the country introduces new ART-based PrEP methods, this pressure will increase. The training of NIMART nurses needs to be scaled up, not only to meet existing demand but also to ensure that PrEP services become fully part of mainstream PHC provision, with enough trained staff to meet this expanding area.

As countries gear up for the introduction of a range of new biomedical HIV prevention products, there are some useful insights which have implications for expanded oral PrEP provision as well as implementation and integration of future products. If PrEP is to be integrated as an integral part of the PHC and HIV care package, rather than as a marginal vertical service, then we need to build on and expand the use of tools already developed to reduce workload. For example, the waiting times and staff shortages could potentially be addressed by digital tools providing information and encouraging informed choice prior to linkage to services or at the service, thereby contributing to decreasing provider time whilst ensuring accurate information on choices aligned to lifestyle (36).

On a more individual level, health providers play a vital role throughout the PrEP journey taken by AGYW, and their attitudes have the potential to promote or hinder access to these services. What we do know is that a substantial proportion of AGYW do not persist with PrEP, and strategies are needed to help them assess their level of risk (and therefore their need for PrEP), and to deal with the everyday challenges they face in taking PrEP (11, 37). Initiation, continuation and effective use of PrEP may be affected by health providers’ lack of evidence-based knowledge about PrEP and reticence about discussing sexual risk (38, 39). Training needs to equip health providers with a solid understanding of how PrEP works, and the confidence to initiate clients onto PrEP and motivate them to continue with this method while their risk remains high. This in turn requires an attitude that holds PrEP in positive regard for what it may offer to young people—which makes our findings on judgemental attitudes about adolescent sexuality very concerning. Such attitudes have a real potential to undermine promotion and delivery of PrEP to young people in South Africa.

To counterbalance these negative views, healthcare worker training needs to explore real-life examples of how stereotypes and prejudicial thinking impact on service delivery and build skills to promote self-care with young clients (40). This could be done through unpacking how PrEP and other HIV prevention options provide tools for young people to take responsibility for their own health and how self-care can contribute to resilience and self-efficacy within an assets based paradigm (41–43). In addition, healthcare workers need to be equipped with a deeper awareness of and sensitivity to the complex contextual dynamics at play for AGYW in terms of gender norms in relationships that set up double standards for them and severely restrict their agency (4, 5, 44). Understanding patterns of sexual behaviour and how these may create barriers to effective PrEP use needs to become an integral part of provider training and guide the messaging conveyed to AGYW (11, 45, 46).

The design and framework for youth-sensitive and youth-responsive services need to consciously factor in the specific needs of AGYW seeking PrEP and other SRH services. This means, for example, not only accommodating the needs of learners who can only access facilities after-hours, but also being sensitive to the needs of young women with work and/or childcare responsibilities. The gender of peer educators, navigators and health promoters should also be taken into account, with due sensitivity to the preference of AGYW wherever possible (17, 28, 47). Alongside training, there is a need for support mechanisms to allow health providers to discuss challenges they grapple with in providing services to young people. Both training and support requires that staff perceptions are addressed in a more deliberate manner, and not as a one-off event but re-visited over time.

The belief that PrEP use will encourage sexual disinhibition, thereby increasing unplanned pregnancies, HIV and STIs, was voiced by a number of healthcare workers in our study and has similarly been documented in other settings (22, 48, 49). Finding ways to deal with judgemental and sometimes prejudicial attitudes is important, including looking at replacing personally held views with evidence, as well as other approaches such as motivational counselling (50) and values clarification and attitude transformation processes (51). In a US-based study, PrEP providers described how their initial ambiguity about PrEP—based partly on a concern about risk compensation—evolved over time as they came to understand their role as helping clients to make informed decisions about their sexual behaviour and use of HIV prevention methods (52). It is possible that such shifts in attitude may be hastened by greater and more meaningful participation of young people in the design and implementation of health services, together with the promotion of dialogues between young people, health providers and programme planners. This needs to take place with a recognition and understanding of young people's rights, within the framework of accessible and acceptable health care (10), and be at the centre of PrEP and SRH service planning and implementation (19).

Finally, while these implications pertain to both Project PrEP and the expanded provision of PrEP in public health facilities in South Africa, the findings highlight the following lessons for Project PrEP: the importance of training including greater gender awareness of AGYWs’ prevention needs; the need to proactively problem solve and strategize with the Department of Health with regards to expanded NIMART and PrEP training; and a need for more engagement with management and frontline health workers concerning the integration of service provision into the life and systems within a clinic. The latter point will become even more important as new PrEP methods are introduced.

There were limitations to the study. The qualitative data analysed here were obtained using a structured survey format, which—in spite of training—may have reduced interviewers’ probing of superficial or incomplete responses. High staff turnover in study sites made it difficult to re-interview participants, as originally planned, limiting our ability to track whether perspectives shifted once health providers had direct experience of PrEP delivery.

## Conclusion

Health providers play a critical role throughout young clients’ PrEP journey, and their attitudes have the potential to influence access, health seeking behaviour, effective method utilisation, and ultimately SRH and HIV health outcomes. The insights gained from this paper underscore the importance of firstly, providing channels to explore health providers attitudes, misgivings and misperceptions and consult about implementation design; secondly, ensuring training includes a purposeful, focussed section on attitudes and values, and sensitisation with all staff clients encounter; thirdly, for programme planners and implementers to engage with managers and health providers about structuring and adapting health systems to support HIV prevention and SRH integration, and for integration to be cross cutting—including HIV and SRH, and the embracing of HIV prevention as an integral part of PHC service provision; and fourthly, providing opportunities for the meaningful participation of young people, feedback mechanisms, and health provider-client dialogues. These are important insights to guide the introduction of new HIV prevention methods as they are introduced into the future.

## Data Availability

The datasets presented in this article are not readily available due to the conditions of the ethics approval of the study. Requests to access the datasets should be directed to the corresponding author.
